# alchemlyb: the simple alchemistry library

**DOI:** 10.21105/joss.06934

**Published:** 2024-09-26

**Authors:** Zhiyi Wu, David L. Dotson, Irfan Alibay, Bryce K. Allen, Mohammad Soroush Barhaghi, Jérôme Hénin, Thomas T. Joseph, Ian M. Kenney, Hyungro Lee, Haoxi Li, Victoria Lim, Shuai Liu, Domenico Marson, Pascal T. Merz, Alexander Schlaich, David Mobley, Michael R. Shirts, Oliver Beckstein

**Affiliations:** 1 Exscientia plc, Schroedinger Building, Oxford, United Kingdom; 2 Department of Physics, Arizona State University, Tempe, Arizona, United States of America; 3 Datryllic LLC, Phoenix, Arizona, United States of America (present affiliation); 4 Open Free Energy, Open Molecular Software Foundation, Davis, California, United States; 5 Differentiated Therapeutics, San Diego, CA; 6 Department of Chemical Engineering and Materials Science, Wayne State University, Detroit, Michigan, United States of America; 7 Université Paris Cité, CNRS, Laboratoire de Biochimie Théorique, Paris, France; 8 Department of Anesthesiology and Critical Care, Perelman School of Medicine, University of Pennsylvania, Philadelphia, Pennsylvania, United States of America; 9 Pacific Northwest National Laboratory, Richland, Washington, United States of America; 10 UNC Eshelman School of Pharmacy, University of North Carolina, Chapel Hill, NC, United States of America; 11 Departments of Pharmaceutical Sciences and Chemistry, University of California Irvine, Irvine, California, United States of America; 12 Silicon Therapeutics LLC, Boston, United States of America; 13 Molecular Biology and Nanotechnology Laboratory (MolBNL@UniTS), DEA, University of Trieste, Trieste, Italy; 14 PM Scientific Consulting, Basel, Switzerland; 15 Stuttgart Center for Simulation Science (SC SimTech) & Institute for Computational Physics, University of Stuttgart, Stuttgart, Germany; 16 University of Colorado Boulder, Boulder, Colorado, United States of America; 17 Center for Biological Physics, Arizona State University, Tempe, AZ, United States of America

## Abstract

*alchemlyb* is an open-source Python software package for the analysis of alchemical free energy calculations, an important method in computational chemistry and biology, most notably in the field of drug discovery (Merz et al., 2010). Its functionality contains individual composable building blocks for all aspects of a full typical free energy analysis workflow, starting with the extraction of raw data from the output of diverse molecular simulation packages, moving on to data preprocessing tasks such as decorrelation of time series, using various estimators to derive free energy estimates from simulation samples, and finally providing quality analysis tools for data convergence checking and visualization. *alchemlyb* also contains high-level end-to-end workflows that combine multiple building blocks into a user-friendly analysis pipeline from the initial data input stage to the final results. This workflow functionality enhances accessibility by enabling researchers from diverse scientific backgrounds, and not solely computational chemistry specialists, to use *alchemlyb* effectively.

## Statement of need

In the pharmaceutical sector, computational chemistry techniques are integral for evaluating potential drug compounds based on their protein binding affinity (Deng & Roux, 2009). Notably, absolute binding free energy calculations between proteins and ligands or relative binding affinity of ligands to the same protein are routinely employed for this purpose (Merz et al., 2010). The resultant estimates of these free energies are essential for understanding binding affinity throughout various stages of drug discovery, such as hit identification and lead optimization (Merz et al., 2010). Other free energies extracted from simulations are useful in solution thermodynamics, chemical engineering, environmental science, and material science (Schlaich et al., 2015).

Molecular simulation packages such as GROMACS (Abraham et al., 2015), Amber (Case et al., 2005), NAMD (Phillips et al., 2020), LAMMPS (Thompson et al., 2022), and GOMC (Nejahi et al., 2021) are used to run free energy simulations and many of these packages also contain tools for the subsequent processing of simulation data into free energies. However, there are no standard output formats and analysis tools implement different algorithms for the different stages of the free energy data processing pipeline. Therefore, it is very difficult to analyze data from different simulation packages in a consistent manner. Furthermore, the native analysis tools do not always implement current best practices (Klimovich et al., 2015; Mey et al., 2020) or are out of date. Overall, the coupling between data generation and analysis in most simulation packages hinders seamless collaboration and comparison of results across different implementations of data generation for free energy calculations.

*alchemlyb* addresses this problem by focusing only on the data analysis portion of this process with the goal to provide a unified interface for working with free energy data generated from different software packages. In an initial step data are read from the native package file formats and then organized into a common standard data structure, organized as a *pandas* DataFrame (McKinney, 2010). Functions are provided for pre-processing data by subsampling or decorrelation. Statistical mechanical estimators are available to extract free energies and thermodynamic expectations as well associated metrics of quality; these estimators are implemented as classes with the same API as estimators in scikit-learn (Buitinck et al., 2013; Pedregosa et al., 2011). *alchemlyb* implements modular building blocks to simplify the process of extracting crucial thermodynamic insights from molecular simulations in a uniform manner.

*alchemlyb* succeeds the widely-used but now deprecated alchemical-analysis.py tool (Klimovich et al., 2015), which combined pre-processing, free energy estimation, and plotting in a single script. alchemical-analysis.py was not thoroughly tested and hard to integrate into modern workflows due to its monolithic design, and only supported (now outdated) Python 2. *alchemlyb* improves over its predecessor with a modular, function based design and thorough testing of all components using continuous integration. Thus, *alchemlyb* is a library that enables users to easily use well-tested building blocks within their own tools while additionally providing examples of complete end-to-end workflows. This innovation enables consistent processing of free energy data from diverse simulation packages, facilitating streamlined comparison and combination of results.

Notably, *alchemlyb*’s robust and user-friendly nature has led to its integration into other automated workflow libraries such as BioSimSpace (Hedges et al., 2019) or MDPOW (Fan et al., 2020), demonstrating its accessibility and usability within broader scientific workflows and reinforcing its position as a versatile tool in the field of computational chemistry.

## Background: Alchemical free energy calculations

Free energy differences are fundamental to understand many different processes at the molecular scale, ranging from the binding of drug molecules to their receptor proteins or nucleic acids through the partitioning of molecules into different solvents or phases to the stability of crystals and biomolecules (Deng & Roux, 2009). The calculation of such transfer free energies involves constructing two end states where a target molecule interacts with different environments. For example, in a solvation free energy calculation, in one state (the coupled state) the target molecule interacts with a solvent (in the case of hydration free energies, water), while in the other state (the decoupled state) the ligand has no intermolecular interactions, which mimics the transfer of a ligand from infinite dilution in the solvent to the gas phase. The solvation free energy is then obtained by calculating the free energy difference between these two end states, but it is crucial to ensure sufficient overlap in phase space between the coupled and decoupled states, a condition often challenging to achieve.

Stratified alchemical free energy calculations have emerged as a de-facto standard approach whereby non-physical intermediate states are introduced to bridge between the physical end states of the process (Mey et al., 2020). In such free energy calculations, overlapping states are created by the introduction of a parameter λ that continuously connects the functional form (the Hamiltonian of the system) of the two end-states, resulting in a series of intermediate states each with a different λ value between 0 and 1 and with the physically realizable end states at λ=0 and λ=1. In general, N alchemical parameters are used to describe the alchemical transformation with a parameter vector $\vec{\lambda }=(\lambda_{1},\lambda_{2},\ldots ,\lambda_{N})$, so that $\vec{\lambda }=(0,\;0,\ldots ,0)$ indicates the initial and $\vec{\lambda }=(1,\;1,\ldots ,1)$ the final state. The intermediate states are non-physical but required for converging the calculations. At each $\vec{\lambda }$-value (or “window”), the system configurations are sampled in the relevant thermodynamic ensemble, typically using Molecular Dynamics (MD) or Monte Carlo (MC) simulations, while generating and accumulating free energy data discussed below. Estimators are then applied to these data to compute free energy differences between states, including the difference between the final and initial state, thus yielding the desired free energy difference of the physical process of interest.

## Implementation

### Core design principles

*alchemlyb* is a Python library that seeks to make alchemical free energy calculations easier and less error prone. It includes functionality for parsing data from file formats of widely used simulation packages, subsampling these data, and fitting these data with an estimator to obtain free energies. Functions are simple in usage and pure in scope, and can be chained together to build customized analyses of data while estimators are implemented as classes that follow the tried-and-tested scikit-learn API (Buitinck et al., 2013). General and robust workflows following best practices are also provided, which can be used as reference implementations and examples.

First and foremost, scientific code must be correct and we try to ensure this requirement by following best software engineering practices during development, close to full test coverage of all code in the library (currently 99%), and providing citations to published papers for included algorithms. We use a curated, public data set (*alchemtest*) for automated testing; code in *alchemtest* is published under the open source BSD-3 clause license while all data are included under an open license such as CC0 (public domain) or CC-BY (attribution required).

The guiding design principles are summarized as:

1. Use functions when possible, classes only when necessary (or for estimators, see (2)).
2. For estimators, mimic the object-oriented scikit-learn API as much as possible.
3. Aim for a consistent interface throughout, e.g. all parsers take similar inputs and yield a common set of outputs, using the pandas.DataFrame as the underlying data structure.
4. Have *all* functionality tested.

*alchemlyb* supports recent versions of Python 3 and follows the SPEC 0 (Minimum Supported Dependencies) Scientific Python Ecosystem Coordination community standard for deciding on when to drop support for older versions of Python and dependencies. Releases are numbered following the Semantic Versioning 2.0.0 standard of MAJOR.MINOR.PATCHLEVEL, which ensures that users immediately understand if a release may break backwards compatibility (increase of the major version), adds new features (increase of minor version), or only contains bug fixes or other changes that do not directly affect users. All code is published under the open source BSD-3 clause license.

### Library structure

*alchemlyb* offers specific parsers in alchemlyb.parsing to load raw free energy data from various molecular simulation packages (GROMACS (Abraham et al., 2015), Amber (Case et al., 2005), NAMD (Phillips et al., 2020), and GOMC (Nejahi et al., 2021)) and provides a general structure for implementing parsers for other packages that are not yet supported. The raw data are converted into a standard format as a pandas.DataFrame (McKinney, 2010) and converted from the energy of the software to units of kT where $k=1.380649\times 10^{-23}\;J{\;K}^{-1}$ is Boltzmann’s constant and T is the temperature at which the simulation was performed. Metadata such as T and the energy unit are stored in DataFrame attributes and propagated through *alchemlyb*, which enables seamless unit conversion with functions in the alchemlyb.postprocessing module. Two types of free energy data are considered: Hamiltonian gradients (dHdl,dH/dλ) at all lambda states, suitable for thermodynamic integration (TI) estimators (Kirkwood, 1935), and reduced potential energy differences between lambda states ($u_{-}nk,u_{nk}$), which are used for free energy perturbation (FEP) estimators (Zwanzig, 1954).

Both types of estimators assume uncorrelated samples in order to give unbiased estimates of the uncertainties, which requires subsampling of the raw data. The alchemlyb.preprocessing.subsampling module provides tools for data subsampling based on autocorrelation times (Chodera et al., 2007; Chodera, 2016) as well as simple slicing of the dHdl and u_nk DataFrames.

The two major classes of commonly used estimators are implemented in alchemlyb.estimators. Unlike other components of *alchemlyb* that are implemented as pure functions, estimators are implemented as classes and follow the well-known scikit-learn API (Buitinck et al., 2013) where instantiation sets the parameters (e.g., estimator = MBAR(maximum_iterations=10000)) and calling of the fit() method (e.g., estimator.fit(u_nk)) applies the estimator to the data and populates output attributes of the class; these results attributes are customarily indicated with a trailing underscore (e.g., estimator.delta_f_ for the matrix of free energy differences between all states). In *alchemlyb*, TI (Paliwal & Shirts, 2011) and TI with Gaussian quadrature (Gusev et al., 2023) estimators are implemented in the TI category of estimators (module alchemlyb.estimators.TI). FEP category estimators (module alchemlyb.estimators.FEP) include Bennett Acceptance Ratio (BAR) (Bennett, 1976) and Multistate BAR (MBAR) (Shirts & Chodera, 2008), which are implemented in the *pymbar* package (Shirts & Chodera, 2008) and called from *alchemlyb*.

To evaluate the accuracy of the free energy estimate, *alchemlyb* offers a range of assessment tools. The error of the TI method is correlated with the average curvature (Pham & Shirts, 2011), while the error of FEP estimators depends on the overlap in sampled energy distributions (Pohorille et al., 2010). *alchemlyb* creates visualizations of the smoothness of the integrand for TI estimators and the overlap matrix for FEP estimators, which can be qualitatively and quantitatively analyzed to determine the degree of overlap between simulated alchemical states, and suggest whether additional simulations should be run. For statistical validity, the accumulated samples should be collected from equilibrated simulations and *alchemlyb* contains tools for assessing (alchemlyb.convergence) and plotting (alchemlyb.visualisation) the convergence of the free energy estimate as a function of simulation time (Yang et al., 2004) and means to compute the “fractional equilibration time” (Fan et al., 2020) to detect potentially non-equilibrated data.

**Figure 1:**
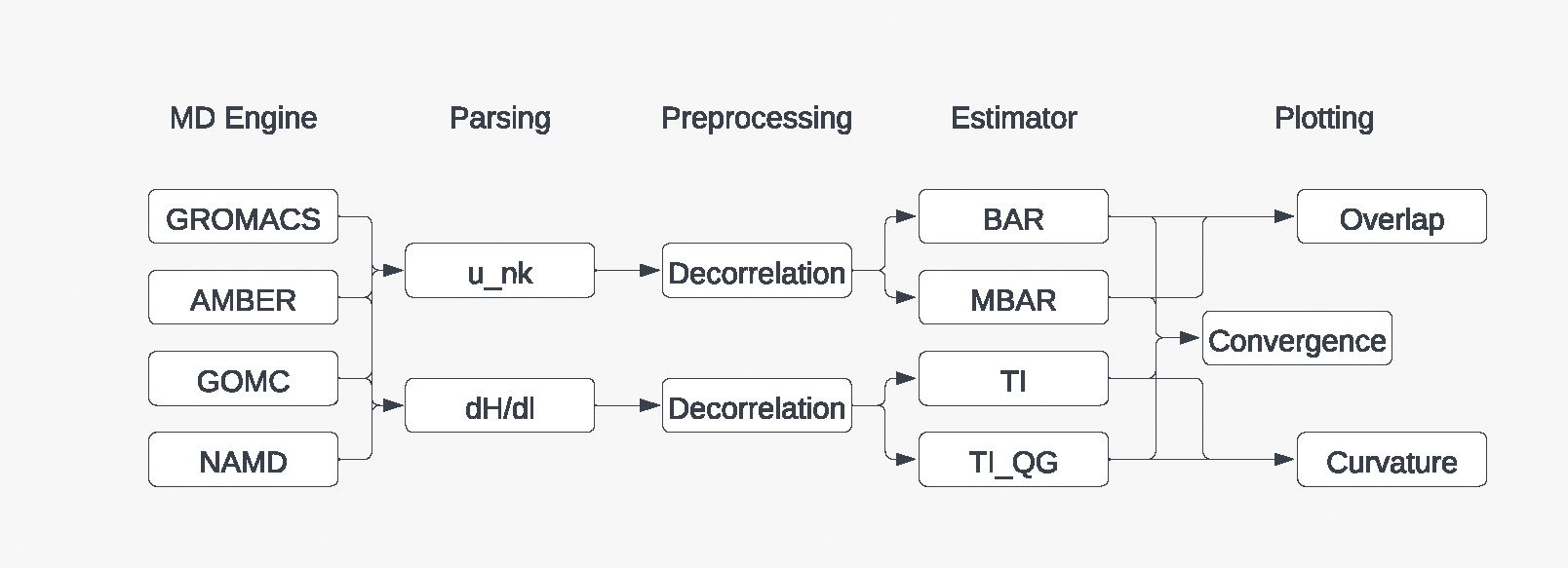
The building blocks of *alchemlyb*. Raw data from simulation packages are parsed into common data structures depending on the free energy quantities, pre-processed, and processed with a free energy estimator. The resulting free energy differences are analyzed for convergence and plotted for quality assessment.

*alchemlyb* offers all these tools as a library for users to customize each stage of the analysis (Figure 1).

### Workflows

The building blocks are sufficient to compute free energies from alchemical free energy simulations and assess their reliability. This functionality is used, for example, by the Streamlined Alchemical Free Energy Perturbation (SAFEP) analysis scripts (Salari et al., 2018; Santiago-McRae et al., 2023).

*alchemlyb* also provides a structure to combine the building blocks into full end-to-end workflows (module alchemlyb.workflows). As an example, the ABFE workflow for absolute binding free energy estimation reads in the raw input data and performs decorrelation, estimation, and quality plotting of the estimate. It can directly estimate quantities such as solvation free energies and makes it easy to calculate more complex quantities such as absolute binding free energies (as the difference between the solvation free energy of the ligand in water and the solvation free energy of the ligand in the protein’s binding pocket).
